# Muscle ruptures in posterior hip dislocation—a case report

**DOI:** 10.1259/bjrcr.20170020

**Published:** 2017-04-13

**Authors:** Florian Alexander Huber, Lena Hirtler, Franz Kainberger

**Affiliations:** ^1^Department of Biomedical Imaging and Image-guided Therapy, Medical University of Vienna, Vienna, Austria; ^2^Division of Anatomy, Center for Anatomy and Cell Biology, Medical University of Vienna, Vienna, Austria

## Abstract

Posterior hip dislocations are the most common luxation types of the hip joint and a well-known and well-described condition. However, we report a case of posterior hip luxation with a series of posttraumatic muscular disorders that were difficult to identify and have not been described previously in scientific literature. We performed clinical and radiological follow-up of an individual for a period of over 19 months post trauma. Informed consent for the anonymized publication of this case was received from the patient. The presented patient is a 20-year-old female, athletic individual in generally good health condition. Our patient suffered from a posterior hip dislocation after a skiing accident on an iced slope. Posttraumatic follow-up was performed owing to persistent moderate hip pain. The patient underwent several experts’ consultations as well as two MRI examinations at 2 months and 19 months after the skiing trauma. Both of the MRIs showed several ruptured parts of the periarticular musculature. At the second MRI, additional compensatory hypertrophy of the piriformis muscle was detected. This report clearly illustrates the importance of profound anatomical knowledge of the surrounding structures of the hip joint, especially as the high psychological strain on the patient could have been reduced by a swifter and appropriate diagnosis.

## Background

Posterior hip dislocation is more common than anterior or central luxation injury and represents 75 to 93% of the patients with dislocations of the respective joint.^1,2^

The mechanism of injury is a direct application of force on the flexed hip, which leads to internal rotation and adduction. The femoral head pops out of the articular cavity either superolaterally or inferolaterally. The typical causes of this pathology are motor vehicle accidents (dashboard injury); other causes are traumas from skiing or American football. Rarely, hip dislocation can also result from massive muscular convulsions due to electrical accidents or severe episodes of seizures.^3,4^

Consequences of hip dislocation typically include injuries of internal structures of the hip joint, proximal fractures of the femur as well as lesions to periarticular soft tissue. The usually involved articular structures are free bodies, tearing of the dorsal labrum of the acetabulum (with or without osseous participation), avulsion of the foveal ligament, rupture of the distal transverse part of the iliofemoral ligament and proximal adhesion of the ischiofemoral ligament with resulting subluxation or wall injuries of the circumflex femoral arteries. This type of injury may lead to posttraumatic avascular necrosis of the femoral head in 10% of these patients. Periarticular injuries typically occur as intramuscular haematomas. Apart from that, 14.5 to 30% of the respective patients ultimately suffer from lesions to the ischiadic nerve, which is located closer to the hip joint in flexion.^2,5–7^

The lateral rotator group, which is adjacent to the dorsal aspect of the joint, is prone to be injured from posterior dislocations. However, the injury of the periarticular muscles, which consist of the internal obturator muscle, the gemelli muscles, the quadratus femoris muscle and the external obturator muscle, have only been mentioned scarcely. Furthermore, the respective literature predominantly describes injuries in the context of postinterventional complications in endoprosthetic surgery.^2,8,9^

We present a case of traumatic hip dislocation. Informed consent for the anonymized publication of this case was received from the patient. Our special focus is on the presentation of the complex periarticular muscular anatomy, which suffered damage and subsequently caused the patient chronic pain.

## Case description

We present a 20-year-old female with athletic constitution in good health condition. She suffered trauma caused by an accident during skiing, where she jumped over a small hill border and landed in a small pit on the slope. The traumatic mechanism of the accident was a ventral-lateral (right side) collision with the solid iced slope in knee and hip flexion at intermediate speed.

According to the patient’s narrative, she was never unconscious, but immediately felt strong pain in her right upper and lower extremities after trauma. Clinical examination of the peripheral extremities showed no signs of neurological or vascular trauma.

### Admission

**Figure 1.**
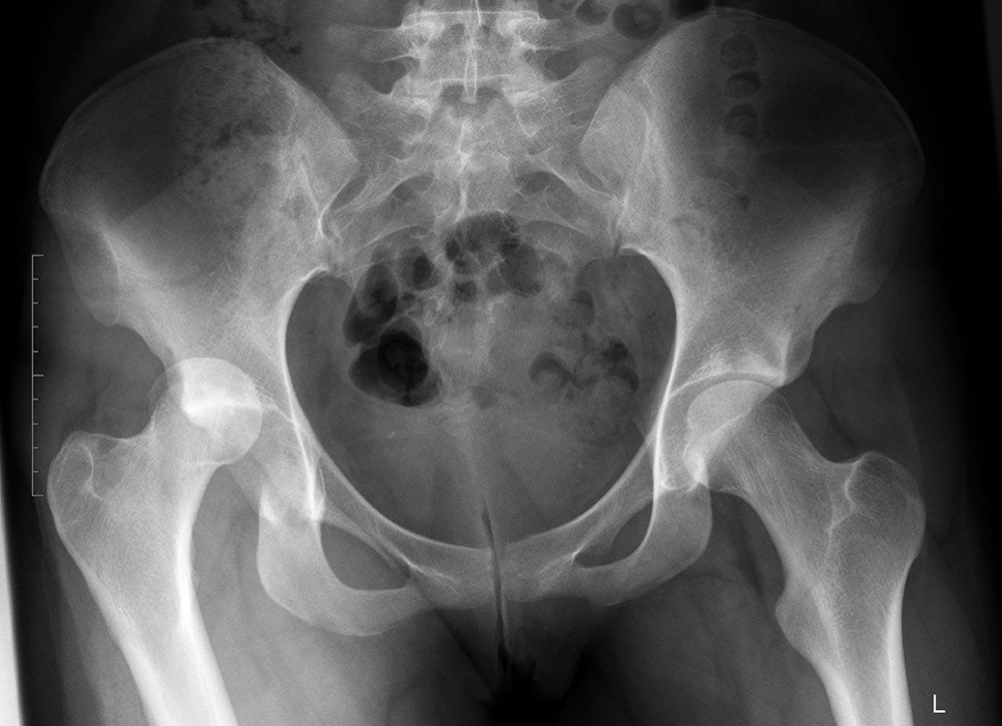
Anterior-posterior X-ray pelvis view, preinterventional, showing clearly the posterior dislocation of the right hip joint, and no visible fracture lines.

**Figure 2.**
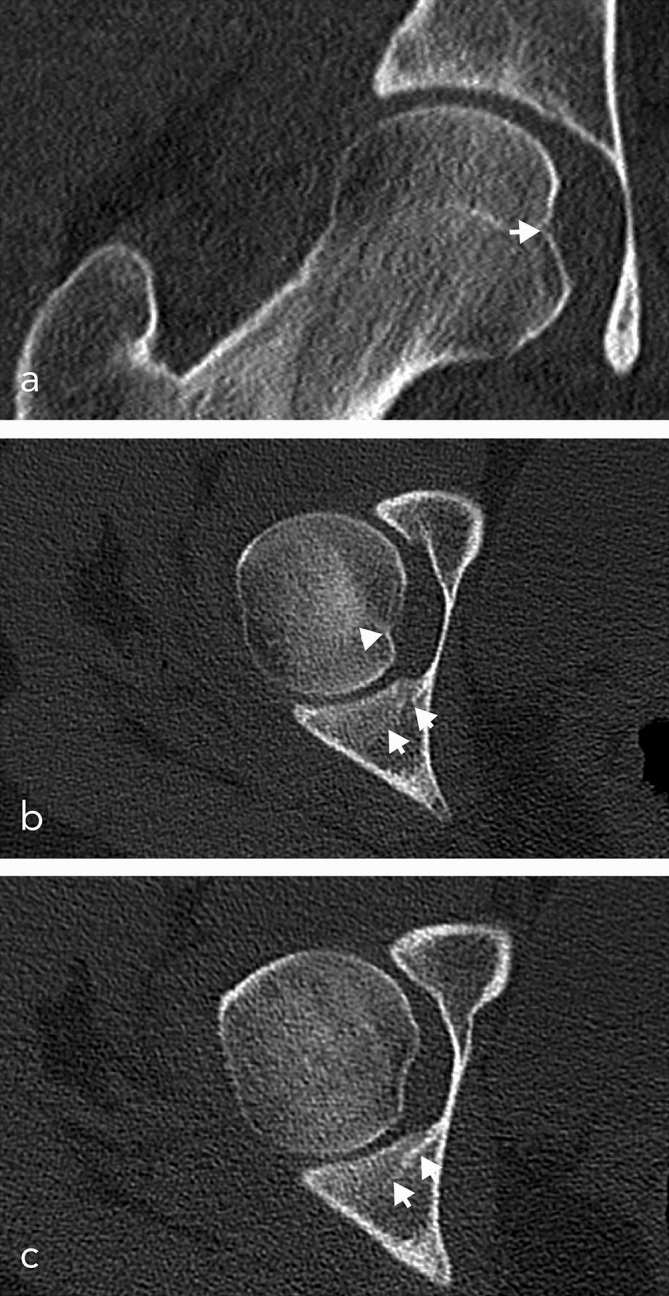
Native CT scans of the hip, postinterventional control: (a) coronal view, (b,c) axial views. (a) Impression fracture of the fovea of the femoral head. (b) Impression fracture of the fovea of the femoral head, fracture line in the posterior column of the acetabulum. (c) Fracture line in the posterior column of the acetabulum.

The patient was hospitalized via flying ambulance to the closest surgical-trauma department shortly after. At admission to the hospital, clinical examination and conservative X-rays in two planes showed a posterior dislocation of the right hip (Thompson–Epstein Grade I), but no signs of fracture. Additionally, X-rays in two perpendicular planes of the right forearm were performed, showing a combined radial and ulnar fracture. The fracture was splinted with split plaster cast of the right arm. The dislocation was treated by joint reduction (Böhler’s technique) under general anaesthesia. The procedure took place around 2 hours after trauma and was performed by a trauma surgeon. A CT scan of the hip was performed afterwards (Figures 1, 2), indicating an acetabular fracture line without any dislocation and consequently needing no further surgical treatment. After one night of medical surveillance, our patient was discharged from hospital with treatment recommendations to use underarm crutches and to consult the nearest trauma department for surgical therapy of the forearm fracture after returning back home. Regarding pain management, the patient received a prescription for non-steroidal anti-inflammatory drugs as prn medication.

Since it was not possible for the patient to handle crutches with her fully casted right arm, she visited the nearest trauma ambulance 3 days after the accident. After denial of two orthopaedic surgeons to perform surgical stabilization of the radial and ulnar fracture, the patient was provided with an arm splint for better handling of the crutches. Owing to unsatisfying success of this option, our patient was advised to hobble. Additionally, she was referred to a physical therapist for remobilization of the injured arm. According to our patient, she was not aware of pain in the hip during this period, but stated that she might have been distracted by the subjectively more severely felt symptoms of her injured arm.

### Two months

**Figure 3.**
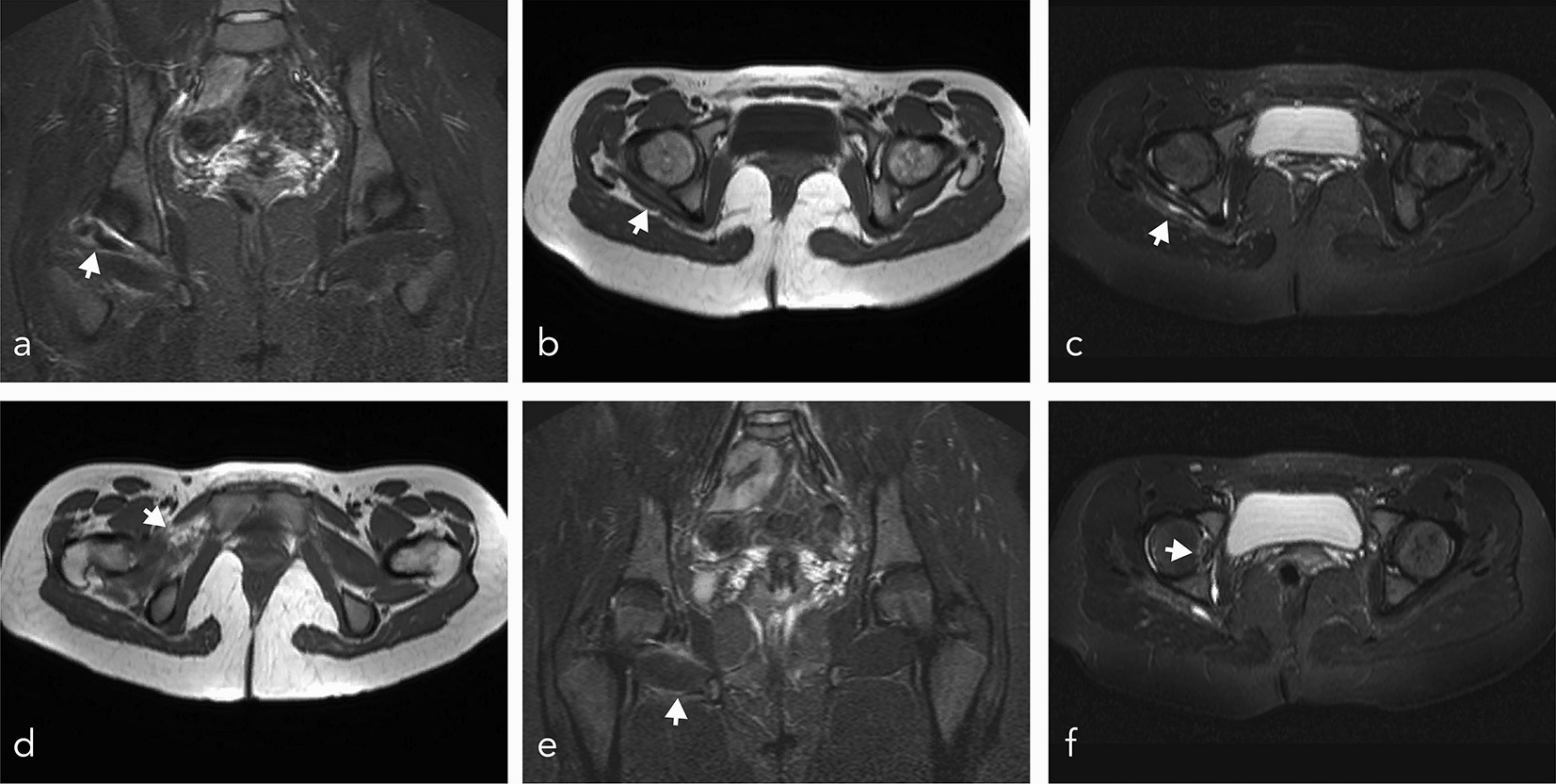
T1 MRI scans, 1 month post trauma, (a, e) coronal short tau inversion recovery sequences, (b, d) axial turbo spin-echo sequence, (c, f) axial short tau inversion recovery sequences. Regular anatomical positioning of the femoral head in the acetabulum, soft tissue oedema in the region of the posterior and inferior periarticular muscles, seen as hyperintensive signal on *T*_2_-weighted images (a, b) retracted muscle belly of the superior gemellus muscle, (c) internal obturator muscle tendon with effusion in the subjacent bursa, (d) tissue oedema in the muscle belly of the external obturator muscle, (e) tissue oedema around the quadratus femoris, (f) small crescent-shaped fissure within the femoral head. No signs of abnormalities of the piriformis muscle, the tensor fascia latae muscle and the gluteal muscles could be detected; there was no fracture of the acetabulum and no signs of intraarticular joint effusion.

A few weeks later, our patient consulted her general practitioner and presented with continuing moderate pain in the right hip. The general practitioner decided to request MRI sequences of the patient’s hip, which showed several trauma-related injuries. On the images (Figure 3), the articulation of the femoral head within the acetabulum was congruent. The posterior and inferior muscles around the joint were altered with severe tissue oedema, visible as hyperintensity on *T*_2_-weighted images. The origin of the superior gemellus muscle was not visible, the muscle belly being retracted and thickened (Figure 3a,b). The obturator internus muscle tendon was surrounded by moderate effusion in the subjacent bursa (Figure 3c). The tendons of the inferior gemellus, the quadratus femoris and the external obturator muscles were normal; there was however severe tissue oedema in the muscle bellies of the quadratus femoris (Figure 3d) and the external obturator muscles (Figure 3e). Piriformis, gluteal and tensor fasciae latae muscles were normal. No abnormalities of vessels or adjacent nerves were observed.

### Nineteen months

**Figure 4.**
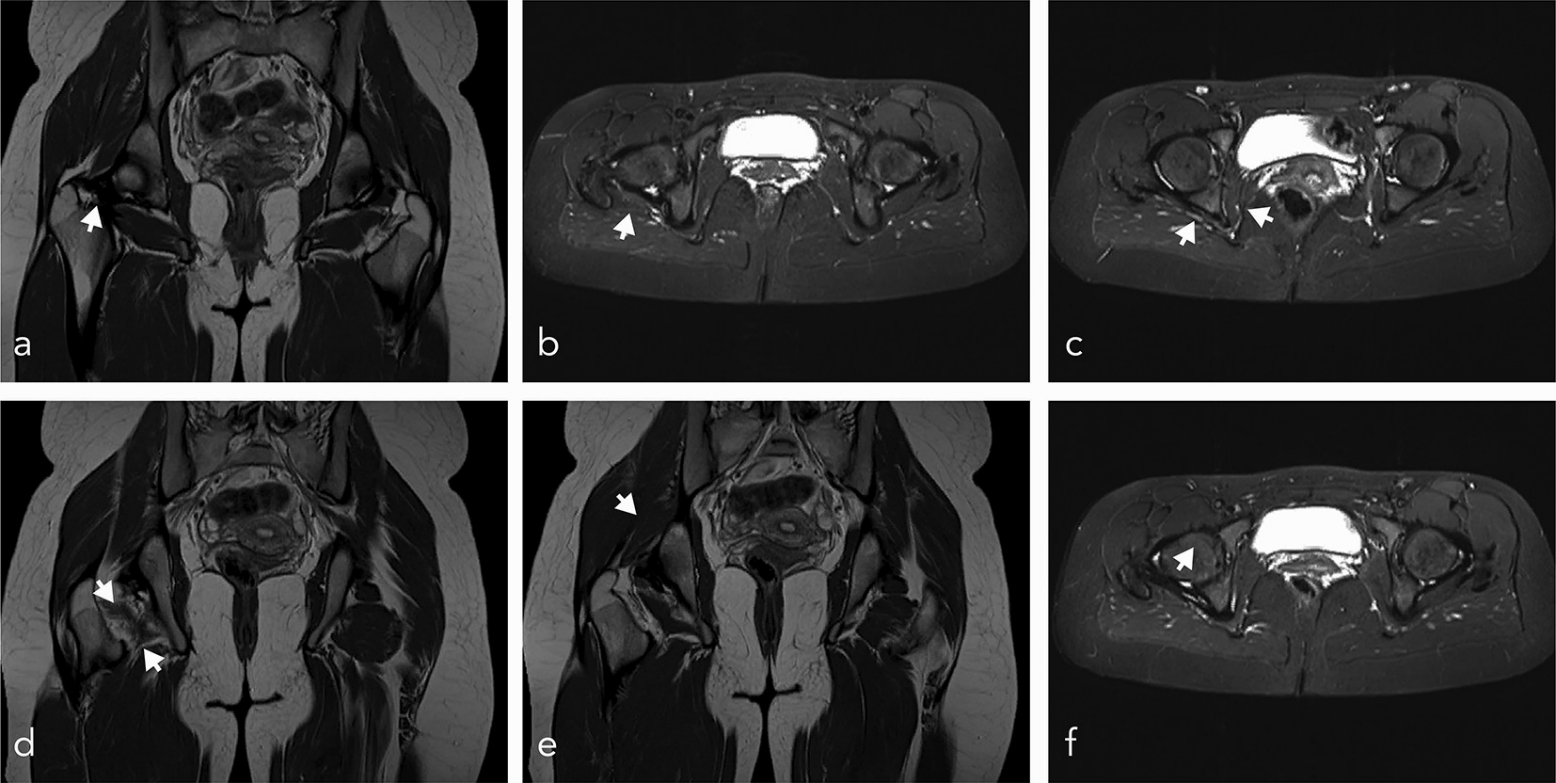
MRI scans, 19 months post trauma, (a, d, e) coronal turbo spin echo proton density sequence, (b, c, f) axial turbo inversion recovery magnitude sequences. (a, b) undulated shape of the tendon gemellus superior muscle, (c) partial rupture of the internal obturator muscle, different than the results from the first MRI scan, (d) scarring in the region of the quadratus femoris muscle, (e) hypertrophy of the piriformis muscle, (f) scarring of the femoral head.

After 19 months of conservative treatment post trauma, our patient was still complaining about mild hip pain. A second MRI was performed to exclude avascular necrosis of the femoral head. The follow-up MRI (Figure 4) showed an undulated shape of the tendon of the superior gemellus muscle (Figure 4 a,b). Although the obturator muscle itself was without pathology in the first MRI, it now was partially ruptured (Figure 4c). There was some scarring around the quadratus femoris muscle (Figure 4d). Scarring of the femoral head was present (Figure 4f). Additionally, we detected hypertrophy of the piriformis muscle (Figure 4e), which was without pathological findings in the first MRI report.

## Outcome

After a surveillance period of over 19 months our follow-up ended with a last patient interview after the reporting of the second MRI of the hip. The patient then still claimed ongoing mild to moderate pain in the right hip, which was worst during longer episodes of upright standing or sitting. Our patient has not felt any symptoms that may have led to a suspected neurological component of the disease mechanism (e.g. sciatic nerve entrapment or piriformis syndrome) at any time. However, the patient felt no more need to continue further treatment besides unsupervised physical therapy owing to the low impact on her activities of daily living.

From the aspect of musculoskeletal imaging we expect several of the reported image findings of the second MRI to be irreversible. First of all, the trophic changes of the piriformis muscle have to be mentioned, which will most probably permanently remain hypertrophic owing to loss of full stability of the triceps coxae muscle. Secondly, the described lesions, especially of the superior gemellus and the obturator internus muscles, are very unlikely to heal anymore after a posttraumatic time period of over 19 months. However, the long-term effects of the described injuries of the periarticular muscles on the patient’s mobility are questionable.

## Discussion

Involvement of the ventral group of the external rotator group has to be suspected in the majority of patients suffering from posterior hip dislocation and was described by Laorr et al.^1^ in 100% of the patients in a case series of 14 individuals with the respective type of injury mechanism. According to Dwyer AJ et al.^10^ muscular injuries can especially be seen in severe types of hip dislocation (Thompson–Epstein classification Type IV, that is, acetabular fracture reaching the ground of the fossa). This also explains the common injury of the capsular ligaments that are adjacent to the respective muscles and support the dorsal stabilization of the hip joint. In particular, the internal obturator muscle is unhitched below its hypomochlion—the lesser sciatic notch—at flexion of the hip. The internal obturator muscle, as well as the neighbouring gemelli muscles, is exposed to enormous tensile load by the dorsally pushing head of the femoral bone. Furthermore, haematomas of the many small surrounding vessels can have an additional effect by means of compression. This is especially caused by venous structures within and lateral to the infrapiriform foramen. These alterations, possibly accompanied by traumatic neuritis of the sciatic nerve, can also occur in dislocations with mild degree of injury (i.e. Thompson–Epstein Type I, presenting with either no or only mild fracture), other than previously described by Laorr et al. and Tannast et al.^1,11^ Therefore, previous terms such as paraarticular haematoma or myositis ossificans should be replaced by more precise reports.

Tthe internal obturator muscle, which is not uncommonly adherent to the gemelli muscles, the gluteus maximus muscle and the quadratus femoris muscle are the strongest external rotators of the hip. Their combined strength exceeds that of all internal rotators. Therefore, the feet are rotated slightly outwards in upright standing position in order to maintain body balance.^12^ Together with the glutei muscles and the big hip flexor, the external rotators are known as crucial elements of the muscular protection of the hip joint.^11^ The long-term complications and symptoms that have been described in this case report may correlate with an insufficiency of the ligamentous apparatus and the muscular cover of the hip.

Nonetheless, muscular injuries could be identified for the first time, which clearly illustrates the crucial value of profound knowledge in musculoskeletal anatomy, even in times of good availability of imaging techniques with high spatial resolution. Additionally, a possible compensation hypertrophy of the dorsal hip muscles could be identified.

## Learning points

1. Comprehensive knowledge of the short muscles of the hip joint is needed to correctly identify lesions in traumatic dislocation of the joint as well as to forestall the mentioned complications in further cases.
2. Special attention to those short muscles should be paid in patients with hip dislocation, as lasting pain may be caused by a rupture in that area. Correct diagnosing of those muscular alterations and their treatment could lead to a better outcome.

## ACKNOWLEDGMENT

The authors would like to thank Prim. Dr. Adolf Georg Lederer for his support in this case report. No grant support was received for planning or conduction of this case report. 

## Consent

Written informed consent for the case to be published (including images, case history and data) was obtained from the patient(s) for publication of this case report, including accompanying images.
